# CXCR4 Antagonism Attenuates the Development of Diabetic Cardiac Fibrosis

**DOI:** 10.1371/journal.pone.0133616

**Published:** 2015-07-27

**Authors:** Po-Yin Chu, Ken Walder, Duncan Horlock, David Williams, Erin Nelson, Melissa Byrne, Karin Jandeleit-Dahm, Paul Zimmet, David M. Kaye

**Affiliations:** 1 Heart Failure Research Group, Baker IDI Heart and Diabetes Research Institute, Melbourne, Australia; 2 Metabolic Research Unit, School of Medicine, Deakin University, Waurn Ponds, Victoria, Australia; 3 Diabetes Complications Division, Baker IDI Heart and Diabetes Research Institute, Melbourne, Australia; 4 Clinical Diabetes and Epidemiology Department, Baker IDI Heart and Diabetes Research Institute, Melbourne, Australia; Indiana University School of Medicine, UNITED STATES

## Abstract

Heart failure (HF) is an increasingly recognized complication of diabetes. Cardiac fibrosis is an important causative mechanism of HF associated with diabetes. Recent data indicate that inflammation may be particularly important in the pathogenesis of cardiovascular fibrosis. We sought to determine the mechanism by which cardiac fibrosis develops and to specifically investigate the role of the CXCR4 axis in this process. Animals with type I diabetes (streptozotocin treated mice) or type II diabetes (Israeli Sand-rats) and controls were randomized to treatment with a CXCR4 antagonist, candesartan or vehicle control. Additional groups of mice also underwent bone marrow transplantation (GFP+ donor marrow) to investigate the potential role of bone marrow derived cell mobilization in the pathogenesis of cardiac fibrosis. Both type I and II models of diabetes were accompanied by the development of significant cardiac fibrosis. CXCR4 antagonism markedly reduced cardiac fibrosis in both models of diabetes, similar in magnitude to that seen with candesartan. In contrast to candesartan, the anti-fibrotic actions of CXCR4 antagonism occurred in a blood pressure independent manner. Whilst the induction of diabetes did not increase the overall myocardial burden of GFP+ cells, it was accompanied by an increase in GFP+ cells expressing the fibroblast marker alpha-smooth muscle actin and this was attenuated by CXCR4 antagonism. CXCR4 antagonism was also accompanied by increased levels of circulating regulatory T cells. Taken together the current data indicate that pharmacological inhibition of CXCR4 significantly reduces diabetes induced cardiac fibrosis, providing a potentially important therapeutic approach.

## Introduction

In the setting of the continued rapid rise in the prevalence of obesity, diabetes mellitus has emerged as one of the fastest growing chronic diseases in the world [1]. The number of people with diabetes mellitus has more than doubled over the last few decades. It has become one of the most important public health challenges globally. The incidence of significant morbidity and mortality associated with diabetes is much worse than that in an age-matched healthy population. This largely reflects the well-known presence of a marked increase in cardiovascular disease prevalence [2]. Indeed, up to two-thirds of all cardiovascular disease deaths occur in people with diabetes or pre-diabetes. Whilst, there is a well established linkage between diabetes, obesity and cardiovascular disease, an independent linkage between diabetes and myocardial dysfunction, diabetic cardiomyopathy, is also well recognized [3].

Epidemiological and clinical observations suggest that the myocardium becomes abnormal in the context of diabetes. In particular, it has been previously shown that the prevalence of left ventricular hypertrophy and heart failure is higher in persons with diabetes, even in the absence of hypertension [4–8]. Echocardiographic studies also confirm the presence of abnormal diastolic function in patients with diabetes, again independent of common coexistent risk factors [9, 10]. Beyond the postulated effects of diabetes per se on the myocardium, it is also evident from epidemiologic and experimental studies that the myocardium has a poorer capacity to tolerate ischemic injury. In particular, the incidence of heart failure and total mortality is higher in persons with diabetes following myocardial infarction [11, 12].

From a mechanistic perspective the pathophysiologic changes in the myocardium that contribute to the development of diabetic cardiomyopathy and the enhanced remodelling response remain unclear, thus preventing the application of specific therapy. A range of contributory processes including renin-angiotensin system (RAS) activation, heightened oxidative stress, lipotoxicity, abnormal calcium handling and inflammation have all been described [13–16]. Increased oxidative stress and inflammation are increasingly considered as co-contributors to a range of myocardial abnormalities including myocardial fibrosis associated with heart failure and hypertension, possibly driven by the RAS or aldosterone [17]. In this setting, we recently showed that stromal derived factor-1 (SDF-1) and its cognate receptor, CXCR4, may play a critical role in the development of diffuse myocardial fibrosis in a dexoycorticosterone acetate (DOCA) hypertensive model [18].

Given the potential role of inflammatory cytokines and chemokines, including SDF-1, as mediators of cardiac fibrosis in the diabetic heart we tested the hypothesis that SDF-1 inhibition using a highly selective CXCR4 antagonist would attenuate the development of cardiac fibrosis in experimental models of type 1 and 2 diabetes.

## Materials and Methods

As outlined above, the current study was designed to specifically test the hypothesis that selective antagonism of the SDF-1/CXCR4 axis would ameliorate the development of cardiac fibrosis in experimental type 1 and 2 diabetes. In the present study we compared the effects of CXCR4 antagonism with RAS inhibition with candesartan. In conjunction, we aimed to test the hypothesis that the bone-marrow derived fibrocytic cells are of importance in the pathogenesis of cardiac fibrosis by performing concomitant bone marrow transplantation with donor marrow obtained from GFP positive mice.

### Animals

All experimental protocols were approved by the Alfred Medical Research and Education Precinct (AMREP) Animal Experimentation Ethics Committee or the Deakin University Animal Welfare Committee under the guidelines of the National Medical and Health Research Council of Australia.

To investigate the impact of CXCR4 antagonism in a type 1 diabetic model, male C57BL/6 mice (6 weeks of age) were obtained from the Central Animal Breeding House of the BakerIDI Heart and Diabetes Research Institute. Mice were randomly divided into the following groups: control (citrate buffer injection, vehicle containing mini-osmotic pump,); control+bone marrow transplantation (BMT) (citrate buffer injection, vehicle mini-osmotic pump and bone marrow transplantation); diabetes (streptozotocin (STZ) injection, vehicle mini-osmotic pump,); diabetes+BMT (STZ injection, vehicle mini-osmotic pump and bone marrow transplantation,); diabetes+candesartan (STZ injection, vehicle mini-osmotic pump, candesartan in drinking water) diabetes+BMT+candesartan (STZ injection, vehicle mini-osmotic pump, bone marrow transplantation, candesartan in drinking water,); diabetes+CXCR4 antagonist (STZ injection, CXCR4 antagonist mini-osmotic pump,) and diabetes+BMT+CXCR4 antagonist (STZ injection, CXCR4 antagonist mini-osmotic pump and bone marrow transplantation). Type 1 diabetes was induced by five consecutive daily intraperitoneal STZ injections (55 mg/kg, in 0.1 mol/l citrate buffer, pH 4.5; Sigma Aldrich, St. Louis MO), 4 weeks after bone marrow transplantation in mice receiving bone marrow transplants. Sham mice were administered an equivalent volume of citrate buffer. Saphenous vein blood glucose levels were measured every 2 weeks using a hand-held glucometer (Accu-chek Go; Roche, Basel, Switzerland) and blood glucose levels exceeding 33 mmol/l were considered to indicate the induction of Type I diabetes. Animals were then followed for a period of 8 weeks, to 18 weeks of age, with interventions according to treatment group allocation. Minipumps were implanted under isofluorane (1–4%) anesthesia.

To complement the study in animals with type I diabetes, we also investigated the impact of CXCR4 antagonism in an experimental animal model of type 2 diabetes. This study was conducted in Psammomys obesus (Israeli sand rats), a model of obesity and type 2 diabetes which occurs in the context of insulin resistance together with insulin deficiency [19, 20]. Animals were fed *ad libitum* a standard rodent diet from which 63% of energy was derived from carbohydrate, 25% from protein and 12% from fat (Barastoc, Pakenham, Australia). At 16 weeks of age, male animals were randomly assigned to two treatment groups: vehicle control or CXCR4 antagonist.

### Bone Marrow Transplantation and Fluorescence-Activated Cell Sorting

Bone marrow cells were harvested from 6-week-old GFP transgenic mice. Mice (n = 48, as outlined above) received lethal irradiation with a total dose of 11 Gy (550 rad x 2, separated by 3 hours). The remaining 24 mice did not receive irradiation. Unfractionated GFP^+^ bone marrow cells (5 x 10^6^ cells) were resuspended in serum-free medium (Dulbecco’s modified essential medium containing 0.5% penicillin-streptomycin and 1% glutamine). These cells were injected into the tail vein in a final volume of 0.2 ml. Mice were sacrificed at 8 weeks for assessment of cardiac fibrosis by Masson’s trichrome staining, GFP accumulation in cells, blood pressure and leucocyte population. To assess chimerism, peripheral blood cells were collected from the recipient mice at the time of sacrifice, and the frequency of GFP^+^ cells among peripheral nucleated blood cells was determined by fluorescence-activated cell sorter analysis using a FACScan (BD Biosystems, Franklin Lakes, NJ). Analysis of flow cytometry data was performed using CellQuest Pro software (BD Biosciences, Franklin Lakes, NJ).

Before sacrifice, blood samples were obtained from all mice. Leucocyte count was quantified using a Sysmex (Roche Diagnostics) automated blood analyser. In a separate cohort FACS analysis of peripheral blood was performed in control and AMD3100 treated mice. 0.5–1.0ml of blood was collected from mice by cardiac puncture and suspended in EDTA. Red cells were lysed (BD Pharm Lyse 555899), then remaining cells were pelleted and suspended in FACS buffer. Remaining cells were stained for CD4, CD25 and Foxp3 (eBioscience) according to the manufacturer’s instructions, followed by flow cytometric analysis (BD FACS Canto LSRII flow cytometer).

### Delivery of Candesartan

The angiotensin II receptor antagonist candesartan cilexetil (kindly provided by AstraZeneca, London, UK) was orally administrated in drinking water at daily dose of 10 mg/kg, starting one day before STZ treatment and then continuing through the 8 week treatment period. Candesartan was dissolved with arabic gum in distilled water, and diluted with water to the appropriate concentration. Arabic gum alone dissolved in water was used as a vehicle.

### Delivery of CXCR4 Antagonists

Delivery of the CXCR4 antagonist or corresponding vehicle was achieved using implanted osmotic pumps (Alzet osmotic pump, Model 2004) inserted subcutaneously through a midscapular or flank incision. In the type 1 diabetic mice, the highly selective CXCR4 antagonist, AMD3465 [21] (kindly provided by Dr Geoff Akita, Genzyme Corp, Cambridge, MA, USA), was dissolved in 0.1N sodium bicarbonate solution and delivered at 6 mg/kg/day for 8 weeks. The CXCR4 antagonist, AMD3100 (Tocris Bioscience, Bristol, UK) dissolved in H_2_O, was delivered in the type 2 diabetic sand rat model at a dose of 6 mg/kg per day for 8 weeks. In complementary studies, the effect of CXCR4 antagonism (AMD3100 6mg/kg/d) on regulatory T cell numbers was examined. For these studies, AMD3100 or vehicle was delivered via minipump for a period of one week.

### Blood Pressure and Gross Morphometry

Systolic blood pressure was assessed by a non-invasive tail cuff system in conscious mice at the end of the study. Animals were habituated to the device before measure the blood pressure on five to six occasions over 15 min to enhance accurate measurements. Animals were killed at the end of the experiments by deep anaesthesia using pentoparbitone (200mg/kg ip) and the heart and kidney were rapidly excised, weighed and, immersed in saline on ice before fixing in either 10% formalin in PBS for paraffin sectioning or 4% paraformaldehyde in PBS solution for frozen sectioning.

### Histological Analysis

Ventricular tissue was prepared for paraffin sectioning or embedding in OCT. Four-micron paraffin sections were stained with Masson’s trichrome to evaluate the distribution and localization of collagen. The extent of fibrosis was measured in each of ten randomly chosen fields per animal in perivascular and interstitial areas with ImagePro Plus software (Adept Electronic Solutions Pty Ltd, Moorabin, Australia) using an Olympus BH2 microscope with results expressed as a percentage of blue area in each screen at a magnification of 400x. Perivascular and interstitial collagen volume fraction of the Masson’s trichrome stained tissue were measured separately. All collagen surrounding an intramyocardial coronary artery was considered as perivascular collagen. Vessels that were located in scars were excluded from the analysis. The investigator responsible for the morphometric analysis was blinded as to each experimental group.

Hearts collected for OCT sectioning were first perfused with PBS solution and perfusion-fixed with 4% paraformaldehyde in PBS solution. Sliced hearts were then embedded (OCT compound; Miles Scientific; Naperville, IL) and quickly frozen on dry ice. Cryostat sections (5 μm thick) were stained overnight at 4°C with anti-α-smooth muscle actin (SMA) [clone 1A4; Sigma Aldrich; St. Louis, MO] to evaluate the mobilization, distribution, and localization of GFP^+^ α-SMA^+^ cells. The sections were incubated for 1 hour at room temperature with secondary antibodies that had been conjugated (Alexa Fluor 546, Alexa Fluor 647; Molecular Probes Inc., Eugene, OR, US). The nuclei were stained with Hoechst 33342 (Invitrogen Australia Pty Ltd, Mount Waverley, Australia). Slides were observed under a fluorescent microscope. The GFP signal was confirmed by staining overnight at 4°C with monoclonal (clone 1E4) or polyclonal anti-Green Fluorescent Protein (GFP)(MBL Co., LTD, Naka-ku Nagoya, Japan). Fluorescent images were obtained using Olympus Fluor Image Pro microscope. The quantification of GFP+ cell was carried out under a microscope (magnification x400). Ten high power fields (each 0.5mm^2^) of the heart tissue (left ventricle free wall, interventricular septum and right ventricle free wall) were examined and were used to calculate the number of cells with GFP+ per hpf.

Immunohistochemistry was performed on paraformaldehyde-fixed frozen sections using a biotin-avidin-peroxidase technique and visualized with diaminobenzidine. Serial cryostat sections (6 μm) were cut, air-dried onto Superfrost plus microscope slides (Thermo Fisher Scientific Inc, Erembodegem, Belgium), and fixed in acetone at -20°C for 20 minutes. Sections were pre-incubated with PBS containing 3% hydrogen peroxide to inhibit endogenous peroxidase activity. Rat-anti-mouse CD4 antibody (1:10, clone RM4-5, BD Biosciences, California, USA), was added and incubated overnight at 4°C. After washing three times in PBS, sections were incubated with the appropriate biotinylated secondary antibody (30 minutes, Vector, Burlingame, CA) followed by avidin-biotin-peroxidase complex (Vectastain ABC kit, Vector, Burlingame, CA), with the subsequent antibody detection using diaminobenzidine (Vector, Burlingame, CA). Histological assessment of CD4+ T-lymphocytes abundance was carried at x400 magnification in 10 separate sections per animal.

### Molecular Biological Assays

Real time PCR was performed to determine SDF-1 mRNA expression in the ventricular myocardium. In brief, total RNA was extracted from left ventricular homogenates using the TRIzol (Invitrogen) purification system and reverse transcribed using reverse transcribed with TaqMan reagents (Applied Biosystems). Real time PCR using 25 ng template using an ABI Prism 7700 sequence detection system (Applied Biosystems) was conducted with the primers for mouse SDF-1: (fwd) 5’-AACCCACCATGCTCATCATTC-3’ and (rev): 5’-TTTCAGGGTCATGGAGACAGTCT-3’; CXCR4: (fwd) 5’-CGTCGTGCACAAGTGGATCT-3’, (rev): 5’-GTTCAGGCAACAGTGGAAGAAG-3’, and for the housekeeping gene 18S (fwd: 5’- TTCGAGGCCCTGTAATTGGA-3’, rev: 5’-GCAGCAACTTTAATATACGCTATTGG-3’. Expression levels were determined by calculating the delta Ct value for each reaction.

### Statistical Analyses

Statistical analysis was carried out using the SPSS software (SPSS 17.0 for Windows, SPSS, Chicago, USA). Values are presented as the mean±SEM. Between group comparisons were performed using Student t-test for normally distributed data. Comparisons between 3 or more groups were performed with ANOVA. A p value of <0.05 was considered to be significant.

## Results

### Cardiac fibrosis

**Fig 1:**
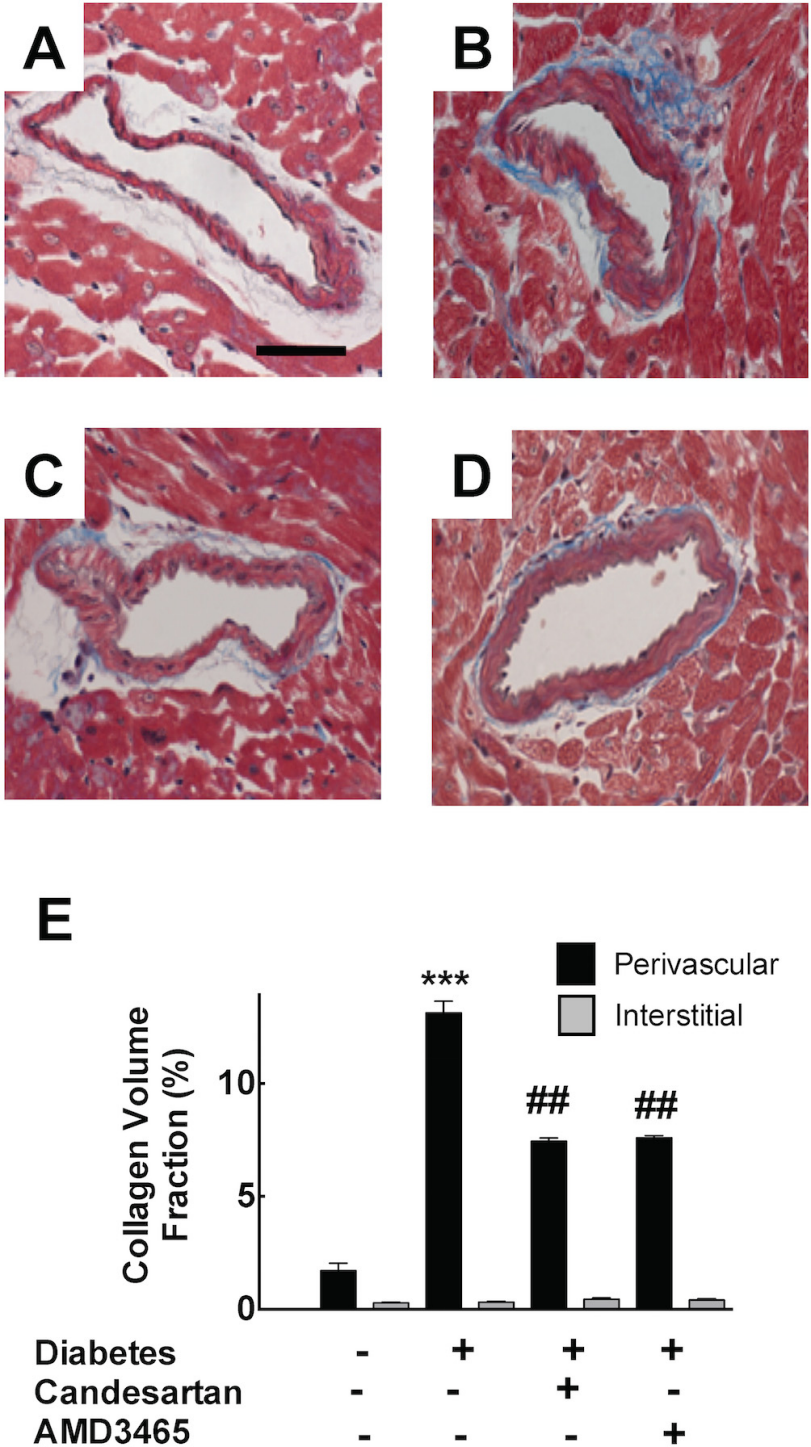
Photomicrographs of Masson’s trichrome stained left ventricular myocardial sections from control mice (A), type I diabetic mice (B), and diabetic mice treated with candesartan (C) or AMD3465 (D). Bar graph (E) represents the extent of perivascular and interstitial fibrosis. *** p<0.001 vs control. ## p<0.01 vs diabetes. Scale Bar 100 μm.

Histological analysis of heart tissue showed that type I diabetes was associated with the development of extensive left ventricular perivascular fibrosis at 8 weeks (Fig 1A–1D) and this was attenuated by both candesartan and the CXCR4 antagonist, AMD3465. By morphometric analysis, there was accompanying evidence of an increase in the heart-to-body weight ratio in type I diabetic mice (Table 1). Quantitative analysis of cardiac fibrosis (Fig 1E) demonstrated that, STZ-treated mice exhibited an increase in the perivascular collagen volume fraction to 13.1±0.5% as compared to 1.7±0.3% in control animals (p<0.001). Candesartan and AMD3465 both significantly reduced the development of perivascular fibrosis to 6.7±0.2% and 7.6±0.1% respectively (both p<0.01). The degree of interstitial fibrosis was relatively low and not different between groups. Bone marrow transplantation did not alter the fibrotic response to diabetes or the response to candesartan and AMD3465 (not shown).

**Table 1:** Gross morphometry in type 1 diabetic mice.

	Control	Diabetes	Diabetes + Candesartan	Diabetes + AMD3465
**Body wt (gm)**	29.3±1.0	22.8±1.0***	26.71.0^##^	26.6±0.5^##^
**Heart wt (mg)**	137±7	114±6*	1267	134±5^#^
**Heart: Body weight ratio** (x10^-3^)	4.7±0.1	5.1±0.1*	4.80.3	5.0±0.2
**Kidney wt (mg)**	224±11	234±12	222±12	233±10

Data are meanSEM (n = 8–12 per gp).

*p<0.05

***p<0.001 vs control

^#^p<0.05

^##^p<0.01 vs diabetes

**Fig 2:**
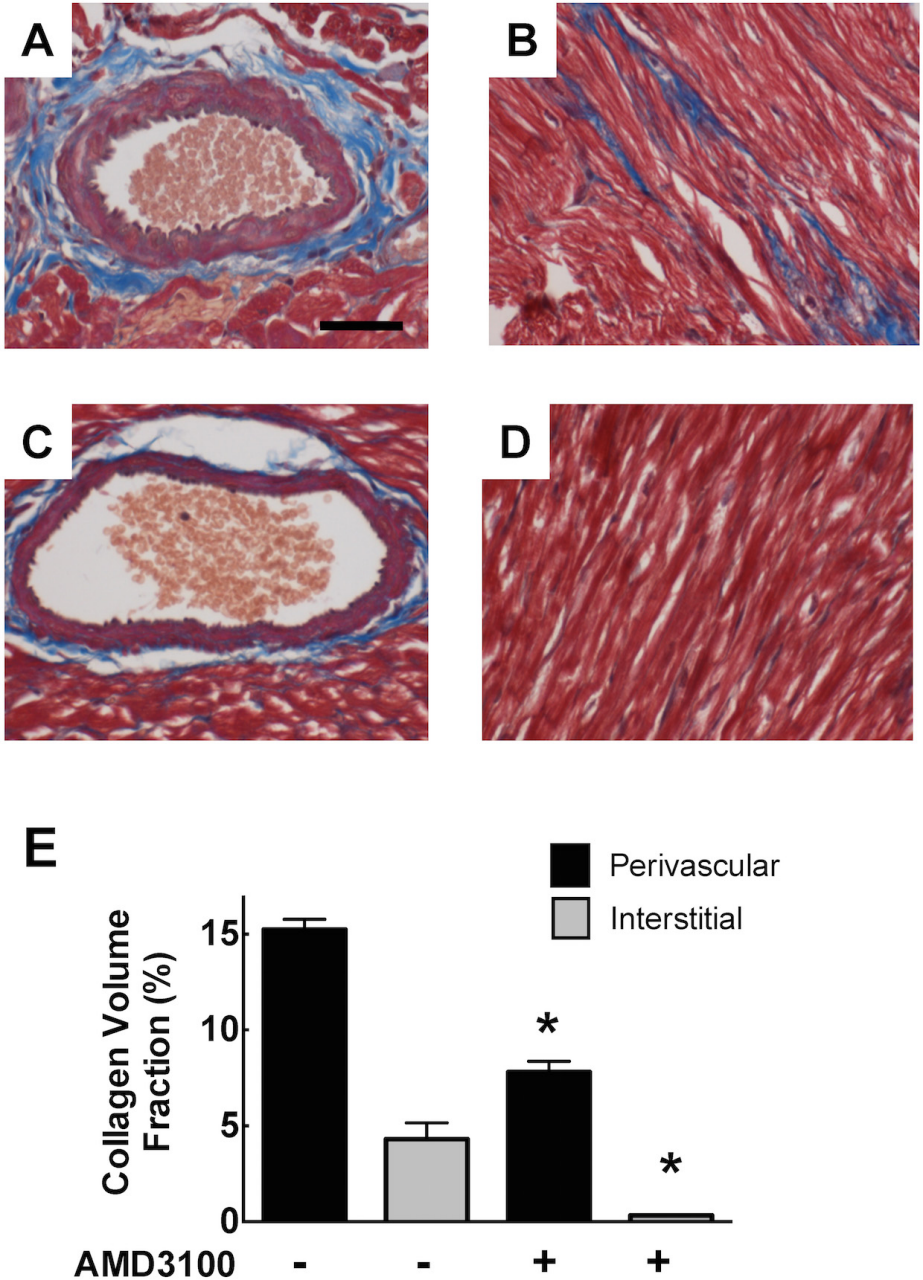
Photomicrographs of Masson’s trichrome stained left ventricular myocardial sections from untreated sand rats (A and B), and rats treated with AMD3100 (C and D). Bar graphs showing the extent of perivascular and interstitial myocardial fibrosis. * p<0.05 vs control. Scale Bar 100 μm.

To complement studies in the type I model, we also investigated the presence of cardiac fibrosis in a spontaneous model of type II diabetes, the Israeli sand rat. Perivascular fibrosis was prominent in these animals under control conditions (15.2±0.5%) and this was accompanied by prominent interstitial fibrosis (7.8±0.6%). Both perivascular and interstitial fibrosis were significantly reduced by the CXCR4 antagonist, AMD3100 at 8 weeks (Fig 2).

### Hemodynamic Effects

**Fig 3:**
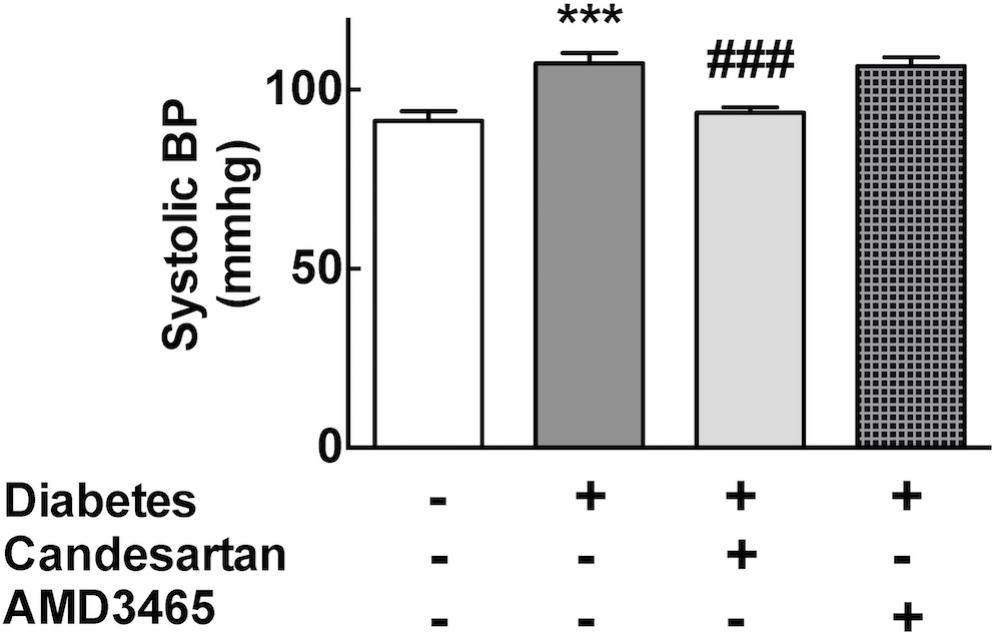
Bar graphs showing tail-cuff blood pressure. *** p<0.001 vs control. ### p<0.01 vs diabetes.

The development of diabetes following STZ administration was associated with a significant increase in systolic blood pressure (diabetes versus sham control: 110±2 versus 95±1 mm Hg, p = 0.001, Fig 3). As expected, in diabetic mice receiving candesartan there was a significant reduction in blood pressure (to 94±2 mm Hg, P<0.001), and this effect was also retained in mice that had undergone GFP bone-marrow transplantation (data not shown). Of note, AMD3465 was without any effect on blood pressure suggesting that the antifibrotic effects of AMD3465 in the heart occur in a blood pressure-independent manner.

### Bone marrow cell recruitment, fibrosis and inflammation

**Fig 4:**
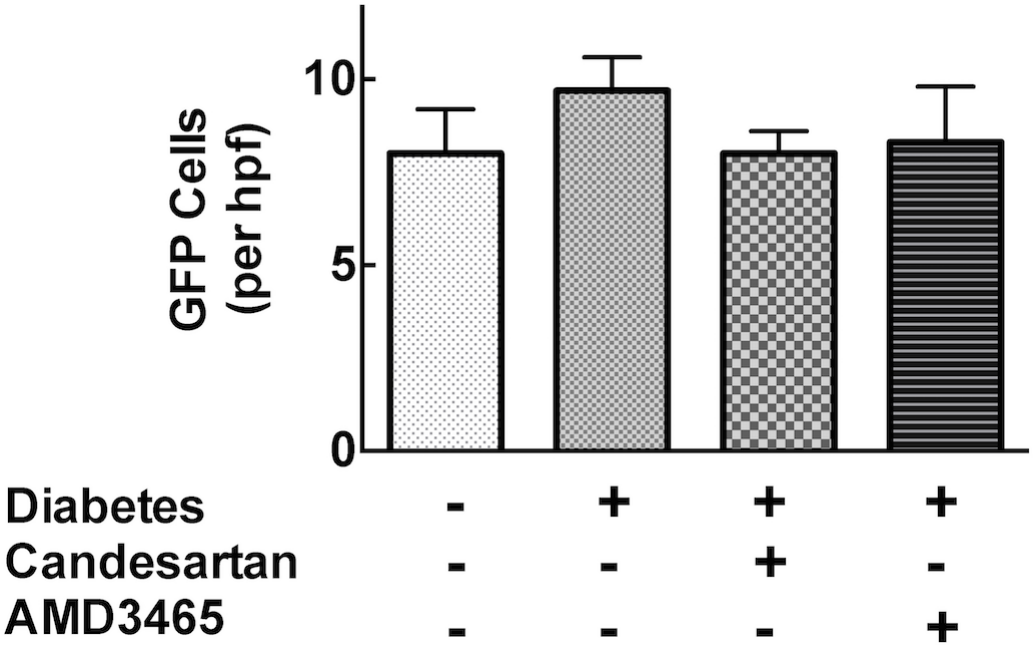
Bar graphs showing the abundance of GFP positive cells per high power field left ventricular myocardium.

To confirm the success of bone marrow transplantation we determined the level of cellular GFP positivity in peripheral blood at 8 weeks. All groups transplanted with GFP+ bone marrow showed high levels (>99%) of expression of GFP+ cells and the efficiency was not influenced by the presence of diabetes or the interventions (data not shown). To investigate whether the recruitment of inflammatory cells contributed to the development of cardiac fibrosis in the diabetic heart, we characterised the extent and nature of infiltrating cells in the heart. As shown in Fig 4, we did not detect any difference in the total abundance of GFP^+^ cells in the left ventricle of diabetic mice compared to those diabetic mice treated with candesartan or AMD3465.

**Fig 5:**
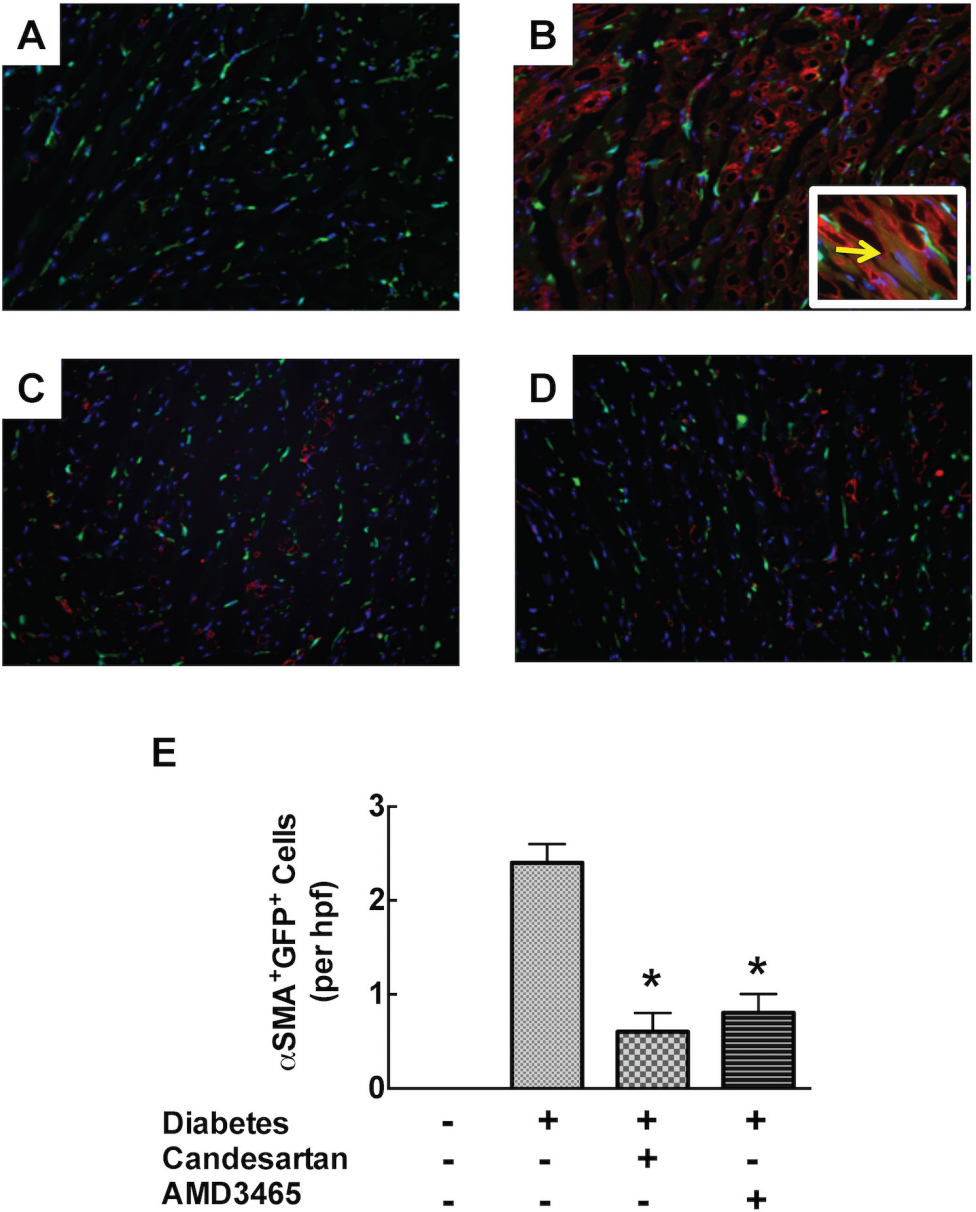
Representative fluorescence photomicrographs demonstrating GFP+ cells (green) and myofibroblasts (alpha smooth muscle actin+ cells, red) in ventricular sections from (A) control, (B) diabetes (C) diabetes+candesartan and (D) diabetes+AMD3465 treated mice. Bar graph (E) represents quantitative data. Photomicrographic insert in 5B demonstrates co-localization of alpha-SMA and GFP (arrow). *p<0.05 vs diabetes.

To determine whether infiltrating GFP+ monocytes and macrophages had contributed to myocardial fibrosis via differentiation into myofibroblasts, we measured also the abundance of cells exhibiting dual GFP and α-SMA positivity. We readily detected the presence of GFP^+^ α-SMA^+^ cells, consistent with presence of bone marrow-derived fibroblasts, in GFP+ marrow transplanted diabetic mice, while in contrast these cells were less evident in diabetic mice receiving either candesartan or AMD3465 (Fig 5). In the STZ model, diabetes was not associated with myocardial CD4^+^T cell accumulation.

Given that STZ exposure induced a strong fibrotic response in the heart, which was attenuated by AMD3465, we assessed the expression of myocardial SDF-1 expression by real time PCR in control and diabetic left ventricle. In this analysis, myocardial SDF-1 mRNA expression was not significantly altered (SDF-1 delta Ct: Diabetes vs Diabetes+AMD3465: 10.4±0.1 vs 10.3±0.5, p = ns). Similarly, the expression of the CXCR4 receptor was unaffected (CXCR4 delta Ct: Diabetes vs Diabetes+AMD3465: 16.3±0.26 16.0±0.26, p = ns).

The effect of CXCR4 antagonism on circulating regulatory T cells (‘Tregs’) was examined in mice treated with AMD3100 or vehicle for a period of one week. FACS analysis demonstrated that CXCR4 antagonism significantly increased the number of peripheral blood CD4^+^Foxp3^+^ Tregs (control vs AMD3100: 7.8±0.3 vs 9.3±0.6%, p<0.01).

## Discussion

Diabetes and its associated metabolic risk factor states are clearly implicated in the pathogenesis of cardiovascular disease. Amongst these disease states, coronary heart disease, stroke and peripheral vascular disease reflect to a substantive degree the effects of accelerated atherosclerosis in combination with dyslipidemia, hypertension and lifestyle factors such as smoking. In regard to the specific manifestations of the cardiac complications of diabetes, while heart attack represents the clinical consequence of atherosclerotic coronary disease, heart failure may result not from injury related to myocardial infarction but also directly as a consequence of diabetes. Patients living with diabetes, have been documented to have an increased risk of developing heart failure [22, 23] and this may be particularly apparent in those with poor glycemic control [24]. In patients with established heart failure, diabetes is an independent risk factor for increased mortality [5, 25, 26].

The relationship between diabetes and changes in left ventricular structure has been well documented. In patients living with type I or type II diabetes an asymptomatic increase in LV mass is a common finding, and this may precede the onset of heart failure [27, 28]. In relation to the clinical development of heart failure, impaired diastolic in particular and systolic performance have both been described in the context of diabetes, independent from other contributory mechanisms [29, 30]. In addition the presence of diastolic dysfunction in the absence of symptoms in patients with diabetes is recognized to be a strong risk factor for the eventual development of overt clinical HF [31].

In the present study, using experimental models of both type I and a model of spontaneous type II diabetes, the Israeli sand-rat, we demonstrated selective antagonism of the SDF-1-CXCR4 pathway reduces myocardial fibrosis, to a degree equivalent to that seen with angiotensin receptor blockade in the absence of an effect on blood pressure. Recently we have showed that CXCR4 antagonism significantly attenuated the induction of cardiac fibrosis, renal fibrosis and left ventricular hypertrophy in the DOCA model of mineralocorticoid excess [18]. Although in that study these observations were associated with marked hypertension, which was mitigated by CXCR4 antagonism, the current study demonstrates that the anti-fibrotic effects of CXCR4 antagonism are not blood pressure dependent. As expected, candesartan did lower blood pressure however the magnitude was modest and likely not contributory to the anti-fibrotic effect. Although recent evidence has suggested that inflammation may be a critical factor in the pathogenesis of fibrosis [15, 32], we did not observe any effect of prolonged CXCR4 antagonism on peripheral blood leukocyte numbers consistent with previous studies of the CXCR4 antagonist AMD3100 [33]. In the present study there were some differences in the magnitude of peri-vascular and interstitial fibrosis between the type I and II animal models, however the effects of CXCR4 antagonism were consistent overall. We did not attempt to determine the specific cause of the differences between the models, however this likely represents the combined effects of a range of genetic and metabolic factors.

Previous studies have demonstrated that diabetes is accompanied by an increase in the vascular expression of SDF-1 [34]. However, the current study does not support the hypothesis that increased myocardial expression of SDF-1 or the CXCR4 receptor occurs in diabetes. Furthermore the study does not indicate that homing of bone marrow derived cells is a significant contributor to cardiac fibrosis. In the present study we did not investigate the secondary effects of CXCR4 antagonism on the relevant SDF1/CXCR4 signalling cascade.

In previous studies of mineralocorticoid excess [35] we showed that CXCR4 antagonism attenuated the accumulation of CD4+ T-lymphocytes. Although, in the current we did not observe significant T cell accumulation, may reflect the relatively late time point of observation, and the less severe nature of the pro-fibrotic stimulus as compared to the mineralocorticoid excess model. The mineralocorticoid model of cardiac fibrosis has been shown to be, at least in part, dependent upon activation of Th17 lymphocytes [36], and the anti-fibrotic effect of mineralocorticoid antagonism by spironolactone is associated with a reduction in Th17 polarization together with an increase in the abundance of CD4+/Foxp3+ Tregs [36]. In the context of experimental models of diabetes, aldosterone levels have also been shown to be elevated during the early phase of the model, and mineralocorticoid antagonism attenuates the development cardiac fibrosis in experimental diabetes [37].

Consistent with growing evidence indicating the importance of the balance between pro-inflammatory Th cells and anti-inflammatory Tregs in the development of cardiac fibrosis, recent studies have demonstrated that the adoptive transfer of Tregs attenuates the development of cardiac fibrosis [38]. In this context, it is also of note that the bone marrow acts as a reservoir for Tregs and these cells may be mobilized by CXCR4 antagonism [39]. In the present study we confirmed the presence of such a mechanism, by demonstrating an increase in the levels of circulating Tregs in the setting of CXCR4 antagonism, and this effect would be expected to lead to an attenuation of activation of Th17 cells.

Taken together, the current data demonstrate that the CXCR4 pathway plays a role in the pathogenesis of cardiac in experimental diabetes. This action appears to occur in a manner independent of blood pressure or the recruitment of inflammatory cells. Further studies of the potential therapeutic utility of CXCR4 antagonism in mitigating or possibly reversing the longer term cardiovascular and renal complications of diabetes should be considered.
